# Book Reviews

**Published:** 1902-02

**Authors:** 


A Text-Book of Diseases of Women. By Charles B. Penrose, M.D., Ph.D., formerly professor of Gynecology in the Univerity of Pennsylvania. Fourth edition, revised. Octavo volume of 439 pages, handsomely illustrated. Philadelphia and London: W. B. Saunders & Co. 1901. [Cloth, $3.75 net.]

Four editions of this book in as many years bespeak the favor it has obtained among both students and physicians. Upon its first appearance it took high place in the literature of gynecology and it has maintained it with increasing force as one edition follows another.

In many parts the book is marked by unusual strength, for example, in injuries to the perineum, displacements of the uterus, diseases of the uterus and appendages, tumors of the uterus and ovaries, and in technique. These are not the precise titles of the sections, but they express in groups the points we wish to emphasize. The author has made such changes in this edition as serve to bring it forward to the present date in its theory and practice. Its continued favor for undergraduates and physicians is well assured.

A Text-Book upon Pathogenic Bacteria. For Students and Physicians. By Joseph McFarland, M. D., Professor of Pathology in the Medico-Chirurgical College, Philadelphia; Pathologist to the Medico-Chirurgical Hospital, Philadelphia; Fellow of the College of Physicians, Philadelphia. With 142 illustrations. Third edition, revised and enlarged. Philadelphia and London: W. B. Saunders & Co. [Price, $3.25 ]

The third edition of Dr. McFarland's book brings his work on the pathogenic bacteria up to date. The chapter on Immunity and susceptibility, which was good in the first edition, has been amplified and extended so as to include even a brief description of Ehrlick's and Wasserman's lateral-chain theory. This chapter is one of the best in the book and can scarcely fail leaving a clear impression of the significance of the theme on the mind of the reader. The chapters on technics and on the individual species of bacteria are fuller than in the previous editions and some new illustrations have been added. Altogether this is an excellent and valuable manual and it is not surprising that it has been successful. It will be even more so, we think, in its revised form.

TWENTIETH ANNUAL REPORT of the State Board of Health of New York. For the year ending December 31, 1899. Transmitted to the legislature February 15, 1900. Albany: James B. Lyon, State Printer. 1901.

This report makes reference to an epidemic of smallpox that began in May, 1898 and crept into the state with a traveling theatrical company at Westfield. This is the outbreak detected by Dr. Ernest Wende, health commissioner of Buffalo, who was called as an expert. Meanwhile the disease had gained such headway that it took more than a year to stamp it out.

The rendering works at Cheektowaga in Erie County are referred to as being in "good shape," which means that they are in a satisfactory sanitary condition, we presume. The report is accompanied with the usual number of papers referring to the conditions in the several important localities of the state; sewerage and sewage disposal claim their full share of attention. After the issuance of one more report, the official acts of the board of health will cease, its duties being merged in the commissioner of health.

